# Cadherin-Dependent Cell Morphology in an Epithelium: Constructing a Quantitative Dynamical Model

**DOI:** 10.1371/journal.pcbi.1002115

**Published:** 2011-07-21

**Authors:** Ian M. Gemp, Richard W. Carthew, Sascha Hilgenfeldt

**Affiliations:** 1 Engineering Sciences and Applied Mathematics, Northwestern University, Evanston, Illinois, United States of America; 2 Department of Molecular Biosciences, Northwestern University, Evanston, Illinois, United States of America; 3 Mechanical Science and Engineering, University of Illinois at Urbana-Champaign, Urbana, Illinois, United States of America; Massachusetts Institute of Technology, United States of America

## Abstract

Cells in the *Drosophila* retina have well-defined morphologies that are attained during tissue morphogenesis. We present a computer simulation of the epithelial tissue in which the global interfacial energy between cells is minimized. Experimental data for both normal cells and mutant cells either lacking or misexpressing the adhesion protein N-cadherin can be explained by a simple model incorporating salient features of morphogenesis that include the timing of N-cadherin expression in cells and its temporal relationship to the remodeling of cell-cell contacts. The simulations reproduce the geometries of wild-type and mutant cells, distinguish features of cadherin dynamics, and emphasize the importance of adhesion protein biogenesis and its timing with respect to cell remodeling. The simulations also indicate that N-cadherin protein is recycled from inactive interfaces to active interfaces, thereby modulating adhesion strengths between cells.

## Author Summary

Tissues are intricate, heterogeneous systems, consisting of individual cells whose shapes and relative positions are of great importance to the tissue's function, as well as to its formation during morphogenesis. To make progress in our understanding of the formation of organs, their malfunction, and their therapeutic replacement in regenerative medicine, it is crucial to elucidate the connection between shape and function. We have developed a quantitative mechanical model of an epithelial tissue, the retina of *Drosophila*, and compare the modeling results with experimental data. The model successfully predicts shape changes induced by different expression levels of cell-cell adhesion molecules. Furthermore, the model gives new insight into the changes a tissue undergoes during morphogenesis. Comparing simulations and experiments, we are able to accept or reject different hypotheses about morphogenetic dynamics. In this way, we can identify the time course of adhesion molecule synthesis and of cell-cell contact, as well as gain new insight into the regulation of adhesion strength. Given the prominent role of adhesion in wound healing, cancer research, and many other fields, our fundamental work introduces a novel modeling tool of universal applicability and importance.

## Introduction

Tissues in multicellular organisms consist of a variety of cells with specialized functions. The differences between cell types are often manifest in the individual cells' shapes, their relative positions, and their neighbor relations [1]. It has become recognized that mechanical forces within cells control cell shapes and their larger-scale organization within tissues [2], [3], [4]. These forces are generated by specific molecules present within and upon the surfaces of cells, including the actin-myosin cytoskeleton and cell-cell adhesion molecules. A fundamental issue is whether tissue morphology can be described as the sum of cell morphologies, which are individually determined by autonomous force generators. An alternative description of tissue morphology assumes that morphology is passively determined by equilibrium mechanics once a small number of force parameters is established by force-generating molecules within cells. This alternative is rationalized by experimental observations finding that cell packing in the *Drosophila* retina is a consequence of mechanical equilibrium [5].Moreover, retinal tissue morphology can be quantitatively modeled by assuming global minimization of interfacial energies that are established by cellular force-generating molecules [6]. Models of other epithelial tissues using similar methods have also successfully reproduced morphological properties [7], [8], [9], giving credence to the approach.

**Figure 1:**
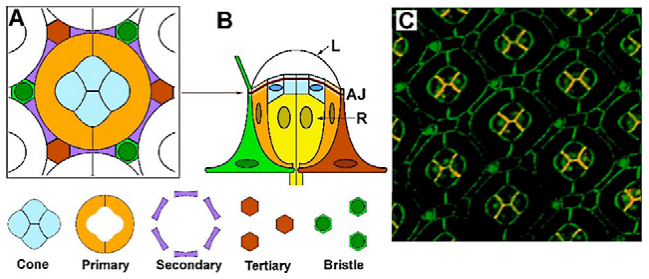
*Drosophila* eye geometry. (**A**) Adherens Junction (AJ) cross section schematic, with the “core” of cone and primary pigment cells and the “frame” of secondary and tertiary/bristle cells. (**B**) Side view of an ommatidium with photoreceptor cells (R) below the AJ and the lens (L) above it. (**C**) Double-stained confocal fluorescence image at the AJ plane of a pupal retina (age 48 h post-pupation). Antibody staining highlights E-cadherin (green) and N-cadherin (red); where the two proteins are co-localized the color appears orange. Note the extreme regularity of the structure.

The *Drosophila* retina is a pseudostratified epithelium containing over 800 repeating units called ommatidia (Fig. 1). Each ommatidium is on average *D*≈9·m across its widest axis, and consists of twenty cells, including eight photoreceptor neurons and twelve accessory cells [10]. Four of these accessory cells (called cone cells) adhere together to form a transparent plate that acts as both the floor of the simple lens and the roof of the underlying pool of photoreceptors. Two primary pigment cells optically insulate each cone-cell group; together with the cone cells, they form the “core” structure of the ommatidium. Secondary and tertiary pigment cells form the ommatidium “frame”. Functionally, the cone cells form an “aperture” for focused light to be transmitted from the lens to the photoreceptors, while the opaque primary pigment cells delineate the aperture stop.

Cells in the retina express two kinds of cell-cell adhesion molecules: E-cadherin and N-cadherin [5], [11]. All cells contain E-cadherin; however, only the four cone cells contain N-cadherin. The cadherin protein molecules become localized in a thin band of lateral cell membrane (*b*≈50 nm wide) corresponding to the adherens junction (AJ), which is the major site of adhesion between cells (Fig. 1B). Cadherins in one membrane bind to cadherins located in the membrane of a neighboring cell across the intercellular gap, affecting the adherence of one cell with its neighbor [5]. The binding interactions appear to be homotypic; E- and N-cadherin molecules do not bind to each other [5], even though heterotypic binding has been found for *Xenopus* cadherins [12]. Intermolecular binding collectively generates adhesion between facing membranes, thereby decreasing the interfacial energy per AJ area and making expansion of the interfacial AJ domain energetically favorable. Membrane that has established such bonds is considered “active”. However, the expansion of one interfacial domain affects other cell-cell interfaces due to constraints on the overall sizes of the AJ membrane domain and cell volume,. Shape changes in one cell induce shape changes in others, and alteration of the elastic energy of the membranes around all cells. Ultimately, the mechanical energy of the entire ommatidium needs to be minimized globally in order to find an equilibrium configuration for the ommatidium [13], [14]. This configuration describes the entire retinal tissue since ommatidia are arranged in the epithelium as identical tiles with six-fold axes of symmetry.

It is well known that cell-cell adhesion can play an important role in cell sorting and morphogenesis of tissues [15], [16], as theoretically described and experimentally demonstrated [17], [18]. More elaborate formalisms have been successfully used to simulate a variety of tissues [19], [20]. Our work indicates that in tissues like the *Drosophila* retina, the role of cadherins goes beyond cell sorting and in fact determines the details of their geometric shapes [6]. In order to understand how cadherin molecules control cell geometry,, one must consider the distribution and dynamic properties of cadherins. In the present work, we apply these considerations to our mechanical model. We then test the fit of various distribution and dynamic models with experimental data for mutants in which certain cells produce altered levels of N-cadherin. We demonstrate (i) that the model describes characteristic shape changes in such mutants, (ii) that the simulations distinguish between different mechanisms of how cadherin levels are attained and controlled, and (iii) that the model, although conceived as an equilibrium tool, incorporates important dynamical features in morphogenesis, such as the temporal sequence of cadherin expression and cell-cell contact remodeling.

## Results

The mechanical model computes cell shapes by the minimization of a mechanical energy functional
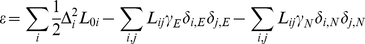
(1)with respect to global shape (deformations of all cells are taken into account), as is described more fully in the Model section. Note that (1) describes a *two-dimensional* energy functional defined in the AJ plane. Since the AJ is only ∼50 nm tall, the structure is two-dimensional to a good approximation, and we can consider mechanical forces and energies in this plane only. Any forces out of plane have to fulfill separate, independent force balances that do not enter into the model.

All cell membranes are assumed to share a uniform stretch modulus. This modulus encompasses energies from actin cytoskeletal contractility and membrane curvature. Two parameters (*γ_E_*, *γ_N_*) encode the adhesion strength of E- and N-cadherin, respectively. These are the only adjustable variables when optimizing lengths of edges between cells (*L_ij_*) and strains on cell circumferences (Δ_i_). We find a unique combination of these dimensionless parameters (*γ_E_*≈0.025, *γ_N_*≈0.032) provides the best fit to experimentally described morphology within experimental error [6].

**Figure 2:**
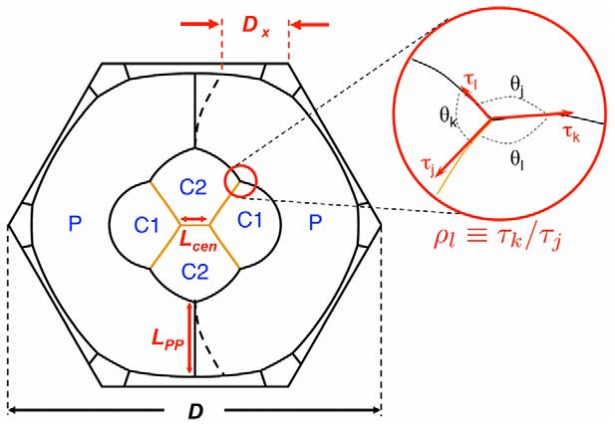
Nomenclature and geometry of the modeled ommatidia. Indicated are the cell types (P,C1,C2), the ommatidial scale *D*, and some of the quantities contributing to the quantification of errors: *L_cen_* and *L_PP_* are two examples of edge length quantities, in the case of an asymmetrically deformed ommatidium (black dashed lines) the asymmetry is quantified using *D*_x_, while errors in angles between edges are expressed in terms of tension ratios. The red circle enlarges one example of a triple junction where the angles *θ_j_* are used to compute the ratios *ρ_j_* of the tensions *τ_k_*, *τ_l_* of the edges adjacent to each angle. Note that *E* cadherin is active on all edges, while on the center edge and the C1C2 edges (orange) *N* cadherin is also active.

Figure 2 shows the modeled AJ structure of such an ommatidium with labels corresponding to the names of each cell. Anterior and posterior cone cells are treated equivalently and are labeled C1; equatorial and polar cone cells are type C2; primary pigment cells have index P. Interfaces are denoted by the names of the cells on either side of a membrane interface, e.g. the interface between a C1 and C2 cell is called a C1C2 edge. We refer to the central C2C2 edge as the center edge. When necessary, we explicitly denote angles by the sequence of cells surrounding them, e.g. a C2/P/C1 angle is the angle between a C2P and a C1P edge.

While the simulation in Fig. 2 fits the geometric features of a normal ommatidium, its two adhesion parameters are open to interpretation. They are proportional to interfacial concentrations of paired E- and N-cadherin molecules, respectively. Although our model uses these concentrations to minimize AJ interfacial energies, how the cells establish and maintain interfacial concentrations of paired molecules is another matter. We have tested a further application of the model by exploring how it is affected by the way in which levels of cadherin protein pairing are regulated. This investigation, as well as the remaining sections of the present work, demonstrate that the energy functional model is capable of addressing and answering questions about important morphogenetic mechanisms.

### N-cadherin dynamics: Destruction vs. recycling

A cell synthesizes cadherin protein in the cytoplasm, and it is transported to the AJ domain of a cell's outer membrane [21], [22]. If a molecule locally pairs with another molecule on a neighboring membrane, then it is stabilized both spatially and temporally [23]. Cell biologists have long known that unpaired cadherin molecules are internalized by endocytosis whereas paired molecules are not [22], [24], [25], [26], [27]. Endocytosis is critical for maintenance of epithelial adhesive integrity [28], [29], [30]. Once internalized, cadherins are either recycled back to the cell surface or trafficked to lysosomes for destruction [31], [32]. Trafficking to lysosomes entails passage through Rab5- and Rab7-enriched compartments [33]. In contrast, recycling back to the AJ occurs by two routes: directly from sorting endosomes or after transport to the recycling endosome [34], [35]. Faced with such disparate alternative pathways, what determines which route is taken after cadherins are internalized? If unpaired cadherins follow a route to destruction, then they do not have an opportunity to redistribute to other membrane domains. If unpaired cadherins are recycled, they are free to distribute on other domains of the membrane, and will accumulate along AJ interfaces with neighboring cells that contain the same cadherin.

**Figure 3:**
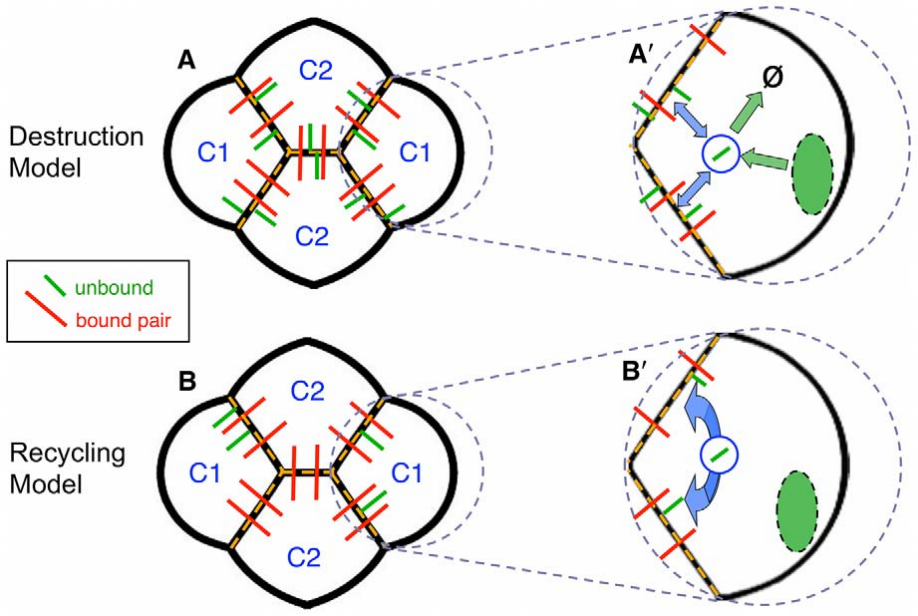
Destruction and Recycling models for N-cadherin distribution. Cone cells with unpaired N-cadherin (green), or paired N-cadherin (red) along active interfaces (dashed orange). (**A**) Distribution and abundance of N-cadherin according to the Destruction Model. Green arrows in the inset **A**′ illustrate that N-cadherin molecules are continually and rapidly synthesized and destroyed (**Ø**). Blue arrows illustrate that unpaired N-cadherin traffics via endosomes (blue) to and from the cell surface. This results in a uniform coverage of active interfaces with an equilibrium distribution of unpaired and paired molecules. (**B**) Distribution and abundance of N-cadherin according to the Recycling Model. The rates of synthesis and destruction of N-cadherin are minor relative to trafficking of N-cadherin to and from the surface (**B**′). Transport of unpaired N-cadherin through endosomes allows continual redistribution of N-cadherin along the cell surface, so that unpaired molecules have multiple opportunities to find partners to pair with. The C2 cells, with the longest active edges, exhaust their unpaired N-cadherin supply. Unpaired molecules remain in the C1 cells.

We formulated two models that simulate these different scenarios for N-cadherin. Both models assume a steady-state concentration of N-cadherin proteins in a cell. The Destruction Model assumes high rates of N-cadherin synthesis and turnover; if unpaired, molecules are rapidly endocytosed and degraded (Fig. 3A). Reaction equilibria are established locally, so that the unpaired concentration determines the concentration of N-cadherin pairs if the neighboring membrane contains the same kind of cadherin. Such local reaction equilibria at interfaces have been described in general terms [36], [37], [38]. The resulting dimensionless binding energy per membrane length is independent of the concentrations on other domains of the AJ in the same cell.

The Recycling Model, by contrast, assumes a low rate of synthesis and turnover of N-cadherin. The cadherin molecules are long-lived and if unpaired, they recycle via endocytosis to and from the AJ. They then redistribute along the AJ in different ways depending on which interfaces are active, i.e. which neighbors express N-cadherin as well (Fig. 3B). In the dimensionless energy functional (1), an N-cadherin binding term along an AJ of length *L* is written as −*γ_N_L* or alternatively −*N_act_ B^dim^*/*E_S_*, where *B^dim^* is the dimensional binding energy of a single N-cadherin pair bond, and *N_act_* is the number of such active bonds along the interface. Thus, if a cell has *N_act_* bonds along a total AJ length *L_act_*, and these bonds are distributed evenly, the effective dimensionless binding strength becomes *γ* = *β N_act_/L_act_*, where we define the constant *β* = *B^dim^*/*E_S_*. Note that *N_act_* is not necessarily the total number of N-cadherin molecules synthesized in a cell, since some molecules may not find a binding partner. Cells contain a defined number N*_0_* of cadherin molecules. We assume this number to be identical for C1 and C2 cells, both for simplicity and because genetic regulation of cadherin expression is not known to differ between cone cells. It is clear, however, that C2 cells have a longer active membrane length than C1 cells (see Figs. 2 and 3). In fact, we can write
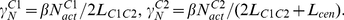
(2)


Only a C2 cell has all of its N-cadherin molecules bound and active (*N_act_^C2^* = *N_0_*), whereas some unbound molecules remain within the C1 cells (Fig. 3B). Quantitatively, the number of those molecules is *N_0_* [1−2*L*_C1C2_/(2*L*_C1C2_+*L*_cen_)]. The binding strength established in the Recycling Model between any two cone cells is thus, *γ_N_* = *βN_0_*/(2*L*_C1C2_+*L*_cen_). Instead of explicitly defining *γ_N_* as a parameter, the Recycling Model implicitly defines it via the parameter *βN_0_* and the self-consistently determined interfacial AJ lengths between cone cells. The C2 cells can be said to be limiting for the process, because all of their N-cadherin is paired, whereas the C1 cells retain some inactive cadherin. In practice, the simulation protocol iteratively adjusts the cadherin strengths and the interface lengths until convergence is reached. In all cases presented here, accurate convergence takes very few iteration steps.

The simulations of normal ommatidia are indistinguishable for the two models, as both of them establish a uniform *γ_N_* along all edges between cone cells. Hence, in order to determine if either model is correct, we turned to situations in which certain cells synthesize less N-cadherin than their neighbors. If asymmetries arise in an ommatidium so that cone cells have different active lengths of AJ with respect to N-cadherin, the two models predict significantly different equilibrium morphologies.

### Simulation of N-cadherin loss

Experiments can be performed in the *Drosophila* eye to create ommatidia where some cells have a normal gene while other cells are missing the gene [5]. Although the cellular composition of such mosaics is generated in a random manner, it is possible to screen through hundreds of ommatidia and find examples where specific cone cells are missing a gene. This was done for the *N-cadherin* gene, and a number of mosaic ommatidia were found. The advantage of working with these mutants is that no additional parameter has to be introduced: normal cells retain normal levels of N-cadherin synthesis, while for mutant cells there can be no binding strength at all.

**Figure 4:**
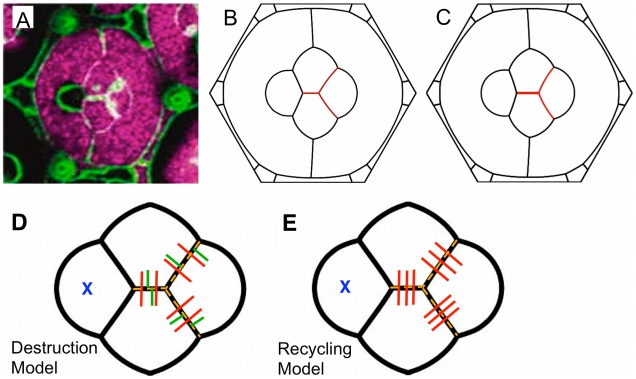
Simulation of ommatidium with one *N-cadherin* mutant cone cell. (**A**) Experimental image of ommatidium with one C1 cell (left) not expressing *N-cadherin*. N-cadherin-producing cone cells are marked in purple, while cone cells not marked purple do not produce N-cadherin. Note that primary pigment cells, whether marked or not, do not normally synthesize N-cadherin. (**B**,**C**) Simulations using the Destruction (**B**) and the Recycling (**C**) Models reflect the general asymmetry and deformation of the ommatidium.. Differences between the models are slight and manifest largely in the center edge length. The width of red active edges is a measure of N-cadherin binding strength in the models. (**D**,**E**) Distribution of N-cadherin according to the Destruction (**D**) and Recycling (**E**) Models. The Destruction Model predicts unaltered densities of N-cadherin molecules and thus unchanged binding strength on the active edges. The Recycling model predicts rearrangement of the unpaired molecules in the C2 cells, leading to increased binding strength across all remaining active edges.

We first examined an ommatidium with only one mutant C1 cone cell (Fig. 4A). Three of the five cone/cone interfaces are active for homophilic N-cadherin binding, while the two interfaces juxtaposed to the mutant cell are seen to be shorter than normal. An overall symmetry breaking of the ommatidium results. Simulations using the Destruction Model resulted in a pattern that reproduces the overall deformation of the ommatidium (Fig. 4B). In the Recycling Model, a more elaborate distribution of binding strength results. The normal C1 cell has a longer active interface to populate than the C2 cells, and consequently becomes limiting for cadherin distribution. However, the surplus N-cadherin molecules in the C2 cells can form active bonds with each other across the center edge. We thus obtain *γ_N_* = 0 on the two inactive interfaces, *γ_N_*≈0.038 on the two active C1C2 edges and *γ_N_*≈0.053 on the center edge, the latter number a much higher binding strength than in the wildtype. The Surface Evolver simulation according to the Recycling Model is shown in Fig. 4C, and is also successful in describing the pattern seen in the experimental image. The differences between Destruction and Recycling simulations are subtle; we quantified the simulation errors using length of the characteristic C1C2 edges on the left side of the ommatidium, together with the angles around them (we use the two tension ratios belonging to the angles P/C1/C2 and P/C2/C1). The definitions of the quantitative individual errors *f_e_* and the total error function *F_e_* are found in the Model section. Table 1 shows that the Recycling Model does better in the total error function *F_e_* (it is smaller by about one third – see Table 1), but there is not enough data to make this finding statistically significant.

**Table 1:** Error measures for N-cadherin knockout mutants.

	*L_C1C2_/D (active)*	*ρ_P/C1/C2_*	*ρ_P/C2/C1_*	*F_e_*
Fig. 4, Experimental	0.0872	0.665	0.865	N/A
Fig. 4, Destruction	0.121	0.607	0.846	0.155
Fig. 4, Recycling	0.117	0.647	0.867	0.113
Fig. 5, Experimental	0.209	1.86	1.74	N/A
Fig. 5, Destruction	0.183	0.92	1.05	0.425
Fig. 5, Recycling	0.217	1.40	1.52	0.0794

**Figure 5:**
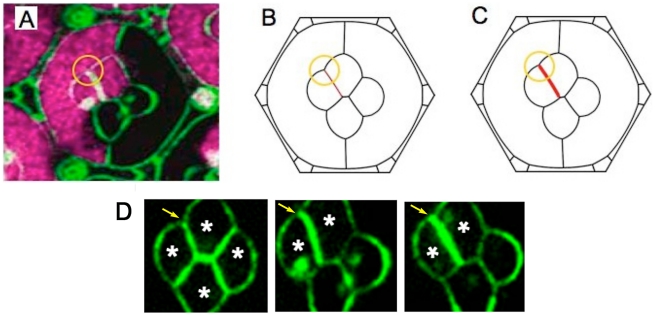
Simulation of ommatidium with two *N-cadherin* mutant cone cells. (**A**) Experimental image of ommatidium with one C1 and one C2 cone cell (right and bottom, respectively) not expressing *N-cadherin* (lack of purple marker). The orange circle indicates the characteristic obtuse C2/P/C1 angle. (**B**) In the Destruction Model simulation, the length of the active edge (red) and C2/P/C1 angle are poorly reproduced. (**C**) The Recycling Model simulation reproduces those quantities significantly better, particularly the C2/P/C1 angle. The thicker red line indicates the predicted increased N-cadherin coverage on the active edge. (**D**) Anti-β-catenin antibody localization in cone cells as visualized by FITC fluorescence. Asterisks mark cells with normal N-cadherin, and unmarked cells lack N-cadherin. The staining of the C1C2 edge from a normal ommatidium (left) is lower than staining on the active C1C2 edges of mosaic ommatidia (center and right), as indicated with arrows. Fluorescence was quantitated on each indicated active C1C2 edge using ImageJ software. The active C1C2 edge fluorescence in the mosaic ommatidia were 1.84- and 2.45-fold greater than the active C1C1 edge in the normal ommatidium.

Therefore, we also analyzed an ommatidium that was missing N-cadherin in the bottom C2 and right C1 cells (Fig. 5A). In the Destruction Model, these two cone cells do not alter the concentration of paired N-cadherin along the single active interface, leading to a normal binding strength *γ_N_*≈0.032, while all other interfaces between cone cells have *γ_N_* = 0. Model simulation resulted in a characteristic asymmetry reflecting what is observed in experiment (Fig. 5B). However, the details of the configuration were poorly reproduced. In particular, the active interface is not long enough and the angle under which it meets with the cone/primary pigment cell edges does not fit the data.

In the Recycling Model, the entire N-cadherin molecule population becomes distributed along the active interface between the two normal cone cells. Not only does this redistribute the cadherin molecules from other interfaces, but even the number of paired N-cadherin molecules is higher because there are no unpaired molecules left over in the C1 cell. As a result, the N-cadherin binding strength along the active interface is enhanced to *βN_0_*/*L*_C1C2_. While *L*_C1C2_ adjusts itself during a Surface Evolver simulation (and becomes longer because of the energetic advantage of long interfaces carrying large cadherin strength), it is clear that this binding strength is much larger than in the Destruction Model. Indeed, the final value from the Surface Evolver simulation is *γ_N_*≈0.081, almost three times the wild-type binding strength (Fig. 5C). The Recycling Model not only simulates the observed asymmetry, but the extreme length and angle associated with the active interface.

The error measures in the simulations with the Recycling Model are all significantly smaller than those with the Destruction Model (Table 1); the total error *F_e_* is more than five times smaller. Thus, a prediction of our mechanical energy model is that cone cells use recycling to redistribute N-cadherin along distinct interfaces. Further evidence supports a recycling/redistribution mechanism in cone cells. In the mosaic experiment shown in Fig. 5, the AJs were visualized using an antibody specific for the β-catenin protein (Fig. 5D). This protein stoichiometrically associates with cadherin protein on the cell membrane, where it helps anchor cadherin to the AJ. Importantly, since β-catenin is produced in vast excess and only bound molecules are stable, its abundance is directly proportional to the abundance of cadherin [22]. The fluorescence intensity of β-catenin staining along C1C2 edges of normal ommatidia is significantly lower than the fluorescence along C1C2 edges of mosaic ommatidia with two mutant cone cells, indicating the presence of much more cadherin on the remaining active edges – a finding that is compatible with the Recycling Model (Fig. 5D), but contradictory to the Destruction Model.

### N-cadherin dynamics: timing and level of expression

The mechanical energy model simulations presented so far implicitly assumed that N-cadherin protein expression is established simultaneously in all four cone cells. Moreover, they assumed equal protein synthesis in all cells. While descriptive fluorescence microscopy supports these assumptions [5], it lacks sufficient resolution and quantitativeness to rigorously demonstrate their veracity. Moreover, the four cone cells do not behave identically from a developmental standpoint. C1 cells begin differentiation several hours before C2 cells, and C1 cells specifically induce cells to a P cell fate whereas C2 cells do not [39], [40]. The mechanical energy model is, however, capable of testing these assumptions in certain mutant ommatidia.

The Destruction Model for N-cadherin would be insensitive to variability of N-cadherin expression since the eventual local equilibria would be established no matter the sequence or level of cadherin synthesis. However, the Recycling Model would be very sensitive to N-cadherin expression. For example, if the C2 cells were to synthesize N-cadherin earlier than the C1 cells and at a stage where the C2-C2 center edge contact is established, all of the N-cadherin from C2 cells would go onto the center edge, for a strength *γ_N_^cen^* = *βN_0_*/*L*_cen_, much greater than observed. There would be no N-cadherin left to bind along the C1C2 edges, rendering the other binding energies *γ_N_^C1C2^* = 0. For similar reasons, the Recycling Model would also be sensitive to changes in the level, rather than the timing, of the expression – changes that the Destruction Model, again, would fail to be affected by.

We therefore examined situations in which certain cells synthesize N-cadherin at different times and levels from normal, in order to (i) further test the Recycling versus Destruction Models, and (ii) establish in what ways morphology is affected by these changes.

### Simulation of N-cadherin misexpression

**Figure 6:**
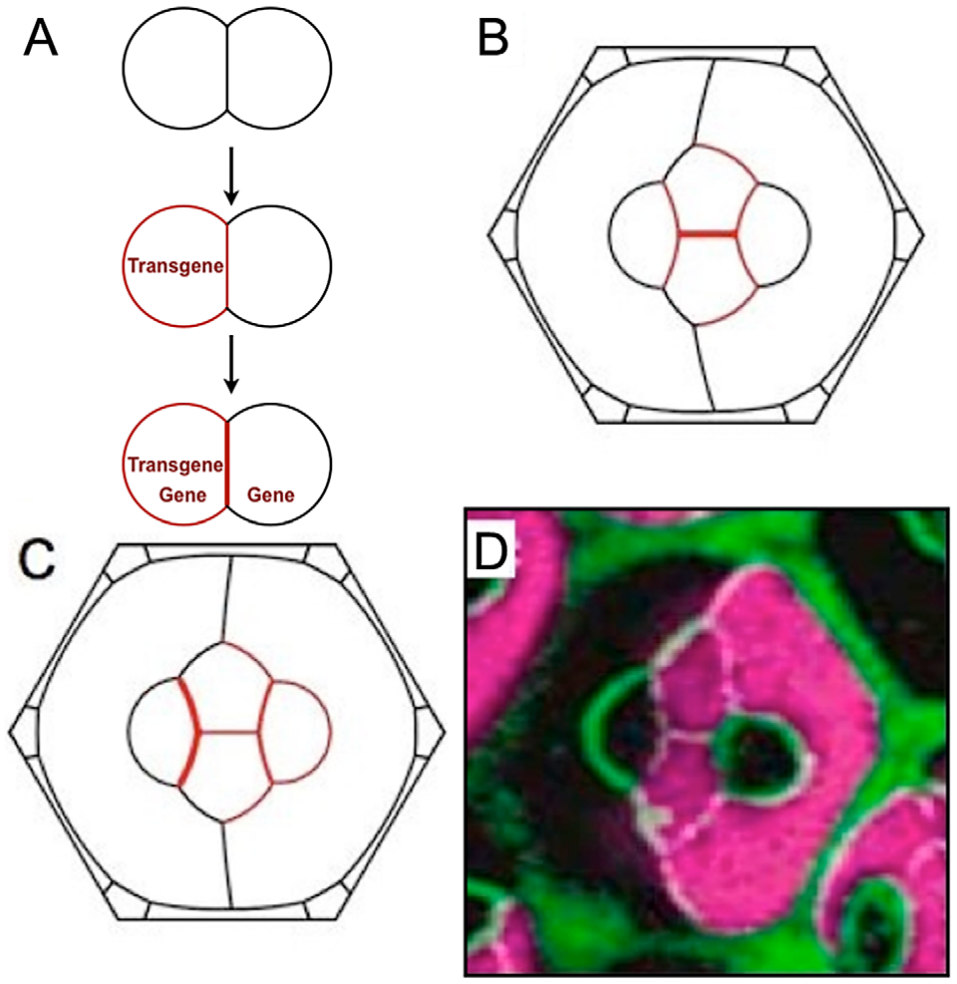
Timing and level of N-cadherin expression affects morphology. (**A**) Order of events (expression) in the misexpression experiment. (**B**) Simulation using the Recycling Model where the *N-cadherin* transgene initiates expression in both C2 cells and one P cell before the endogenous gene begins expression. The level of N-cadherin expression is 90% greater in the C2 cells than C1 cells (*N_+_*/*N_0_* = 0.9). (**C**) Simulation using the Recycling Model as in A but where the transgene and endogenous gene begin expression at the same time, and where the level of N-cadherin expression is 60% greater in C2 cells than C1 cells (*N_+_*/*N_0_* = 0.6). (**D**) Experimental image of ommatidium with the C2 cells and one P cell misexpressing the *N-cadherin* transgene (marked by purple).

We examined mosaic ommatidia in which some cells misexpressed N-cadherin protein. These ommatidia were made by a random genetic switch that activated expression of a *N-cadherin* transgene in certain cells. Since the switch can be triggered in cells irrespective of their identity, pigment cells can potentially express *N-cadherin*. Moreover, cone cells affected by the switch express *N-cadherin* from two sources - the endogenous *N-cadherin* gene and the transgene i.e., the total number of N-cadherin molecules is greater than the normal number *N_0_*. Production of N-cadherin from the transgene is defined as *N_+_*. Switching on of the *N-cadherin* transgene does not occur synchronously with the onset of endogenous *N-cadherin* expression but rather occurs 10 to 30 hours earlier in time [5]. Thus, the transgene not only perturbs the level but also the timing of N-cadherin expression (Fig. 6A).

We modeled an ommatidium that had *N-cadherin* trangene expression in two C2 cells and one P cell. With the Recycling Model, the question of which cell is limiting for N-cadherin depends on the ratio *N_+_/N_0_*. However, except for very extreme ratios, the large active interface of the P cell makes it limiting. The binding strength along the P cell is then *γ_N_^P^* = *βN_+_*/(2*L*_C2P_+*L*_C1P_). The next limiting cell is the right-hand C1 cell, which retains *N_0_*−*L*_C1P_ *γ_N_^P^* molecules, distributed along the C1C2 edges, where the binding strength is *γ_N_^C1C2^* = *β* (*N_0_*−*L*_C1P_ *γ_N_^P^* )/(2*L*_C1C2_). Thus, the overexpressing C2 cells retain a number of N-cadherin molecules given by
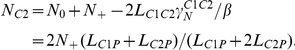



This number is to be distributed along the left-hand C1C2 edges and the center edge. Depending on whether this number is greater or smaller than *N_0_*/2, the left-hand C1 cell or the C2 cells will be limiting.

**Figure 7:**
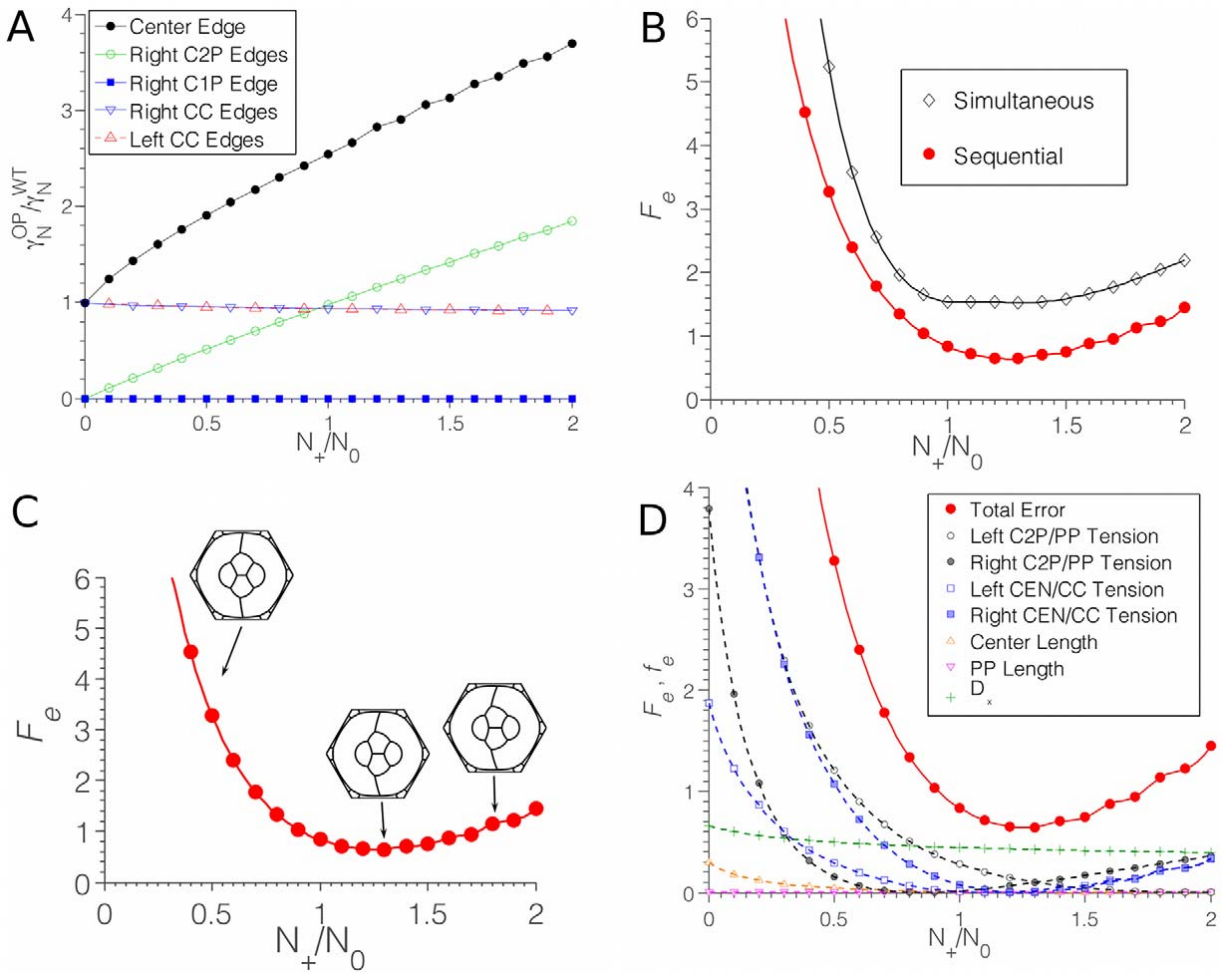
Analysis of misexpression simulations. (**A**) Relative binding strengths on various interfaces as the *N_+_*/*N_0_* ratio varies. Binding strength when N-cadherin misexpressed (OP) was normalized to the wildtype binding strength (WT). (**B**) Total error (values of the total error function *F_e_*) as a function of the *N_+_*/*N_0_* ratio. Red line describes error from simulation in which the transgene and endogenous gene begin expression sequentially. Black line describes simulation in which the two genes begin expression simultaneously. (**C**) The effect of changing *N_+_*/*N_0_* on the shape of the ommatidium in the sequential simulation. For small *N_+_*/*N_0_* (here shown for *N_+_*/*N_0_* = 0.4 on the left), the asymmetry is weak; for large *N_+_*/*N_0_* = 1.8 (right), the differences are more subtle, and the quantitation through *F_e_* is necessary. (**D**) Total error is dominated by contributions *f_e_* from the tension ratios of cone/cone cell edges and C2/P cell edges, with significant contributions from the asymmetry parameter *D*_x_.

Because the transgene is expressed before the endogenous gene, the P cell initially distributes its N-cadherin along the interfaces contacting the two C2 cells also expressing the transgene, so that *γ_N_^P^* = *βN_+_*/(2*L*_C2P_). The remaining *N_+_*/2 molecules in each C2 cell distribute to the center edge with a binding strength of *γ_N_^cen,1^* = *βN_+_*/(2*L*_cen_). Thereafter, the endogenous *N-cadherin* gene is expressed. We assumed that the transgenic N-cadherin would not influence the binding behavior of the endogenous N-cadherin molecules, so that they would distribute similarly (though not identically) to a normal ommatidium. The result of a simulation for *N_+_*/*N_0_* = 0.9 is shown in Fig. 6B. The cadherin binding strengths along different interfaces vary depending on the ratio of *N_+_*/*N_0_*, with very strong binding along the center edge (Fig. 7A). Qualitatively, the simulation matched an observed experimental ommatidium with the same configuration of misexpression (Fig. 6D). The right-hand C1 cell is bulged out with an elongated C1P edge, while the misexpressing P cell has become more compact and rounded, curving the PP edges at the top and bottom of the ommatidium. We quantified the error in the simulation compared to the observed shape to allow for an unbiased comparison. All of the errors were evaluated as a function of the ratio *N_+_/N_0_*. The overall penalty function showed a well-defined minimum at *N_+_/N_0_*≈1.25 (Fig. 7B, C). All in all, we evaluated 15 different error contributions (accounting for the left/right asymmetry, there are 6 different tension ratios, 8 different edge lengths, and the parameter *D_x_* – see the Model section for exact definitions of the error quantities). However, we found that only a handful of these measures contribute significantly to the total error (Fig. 7D). We also tested the shapes and penalty function values assuming a Destruction Model for this example, but total error values were very high in this case (data not shown). Thus, the Recycling Model is strongly supported by simulations of N-cadherin misexpression.

### The importance of N-cadherin timing and cell-cell remodeling

We determined the sensitivity of the Recycling Model to timing of N-cadherin expression, using the example already described. We reran the simulation with the assumption that endogenous and transgenic N-cadherin were expressed simultaneously. This resulted in a different pattern from observed (Fig. 6C). The penalty function for the simulation was greater than the penalty for the simulation with real timing (Fig. 7B). Thus, the Recycling Model predicts that tissue morphogenesis is highly dependent on timing of cadherin expression even if levels are unchanged.

**Figure 8:**
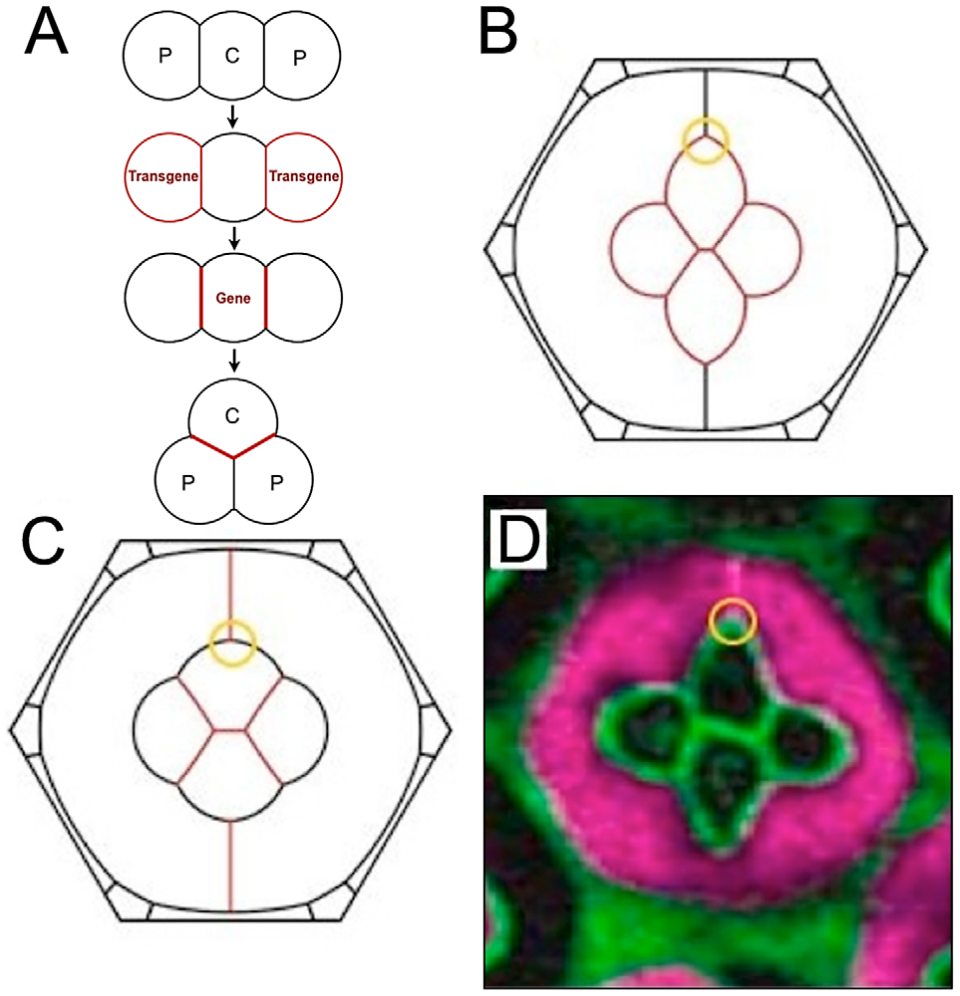
Timing of N-cadherin expression with cell-cell remodeling is important for morphology. (**A**) Order of events (expression and morphogenetic) in the P cell misexpression experiment. (**B**) Simulation in which *N-cadherin* expression in P cells begins before they contact one another. All four cone cells only produce N-cadherin from its endogenous gene. Here N*_+_*/N*_0_* = 1.2, close to the minimum of the total error function. (**C**) Simulation in which *N-cadherin* expression in P cells begins at the same time as they contact one another. For N*_+_*/N*_0_* = 1.0,, the shape compares poorly with observed. This remains true for the entire range of N*_+_*/N*_0_* ratios. (**D**) Experimental image of ommatidium with both P cells misexpressing the *N-cadherin* transgene (marked purple). In all figures, the orange circle marks the characteristic acute angle of the mutant P/C2/P junction.

Our Recycling Model illustrates how gene expression established at different times in different cells can lead to dramatically different morphologies. There is another implication to this; cells frequently change neighbors in a prescribed sequence over time during morphogenesis. Thus, even two cells that initiate N-cadherin expression at the same time might not be able to pair their molecules until such time that they become neighbors. This could have profound consequences on the ultimate distribution of N-cadherin and cell morphologies in a recycling scenario. To test this hypothesis, we exploited the known sequence of neighbor changes that P cells undergo (Fig. 8A). P cells first exclusively contact C1 cells; then P cells form additional contact with C2 cells, and only afterwards do they form contact with each other [10]. In normal ommatidia, this has no consequences for N-cadherin binding, as P cells do not contain N-cadherin.

**Figure 9:**
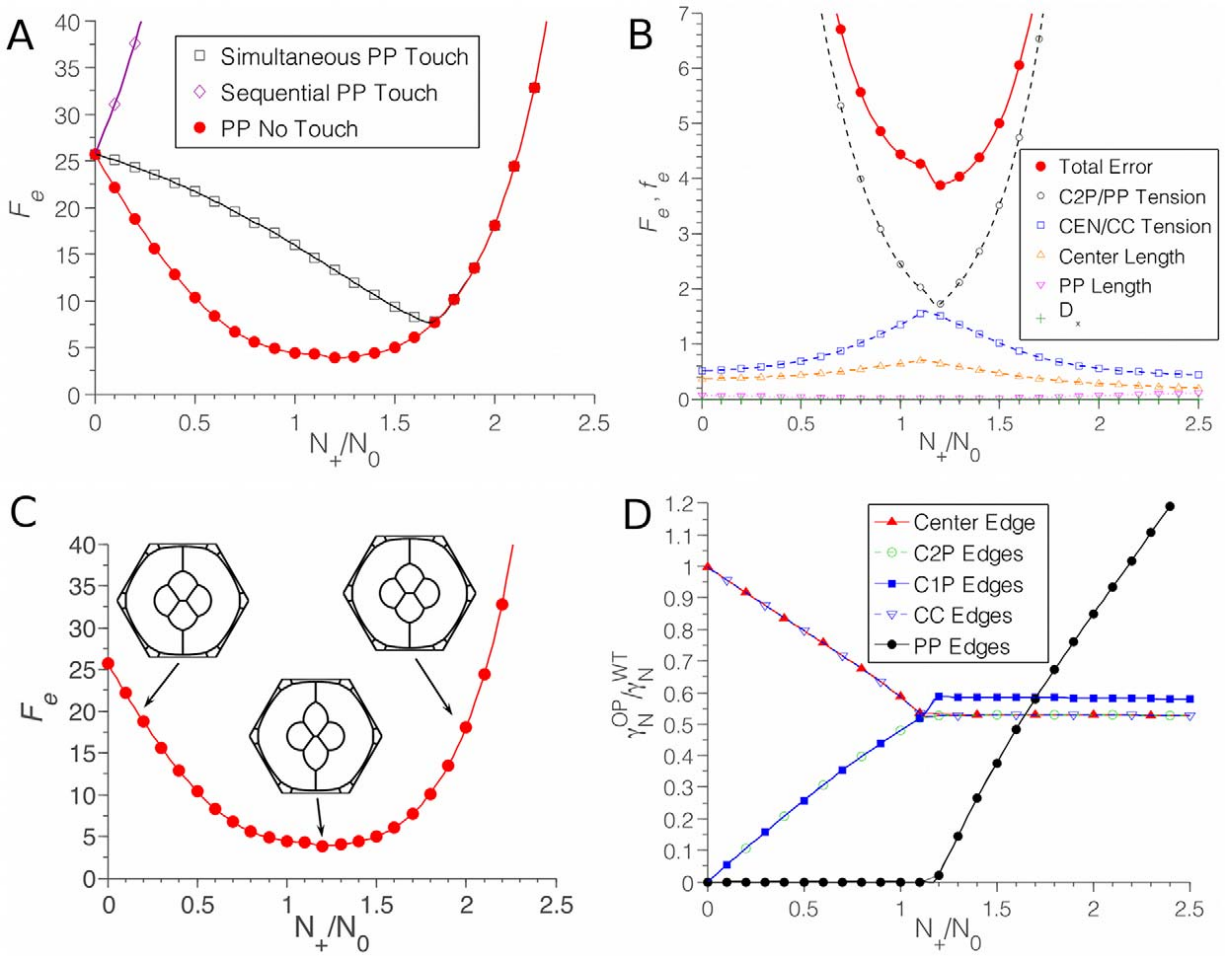
Analysis of P cell misexpression simulations. (**A**) Total error *F_e_* for different simulations as a function of N*_+_*/N*_0_*. The red line describes the simulation error when P cells express *N-cadherin* before cone cells, and P cells do not contact each other until after the onset of N-cadherin expression in cone cells. The purple line describes simulation error when P cells express *N-cadherin* before cone cells but P cells are always in contact with each other. The black line describes simulation error when P cells and cone cells simultaneously begin *N-cadherin* expression, and P cells always are in contact with each other. (**B**) Error contributions to the best-fit model are dominated by the same *f_e_* terms as in the other misexpression simulation, except that the symmetric structure of this mutant has no *D*_x_ contribution to the error. The minimum is located at N*_+_*/N*_0_*≈1.2. (**C**) The effect of N*_+_*/N*_0_* on the shape of the ommatidium, with only values near the error function minimum approximating the observed cruciform mutant shape. (**D**) Binding strengths of N-cadherin on various edges of the structure as N*_+_*/N*_0_* varies. Note the absence of binding on PP edges for N*_+_*/N*_0_*<1.2.

We modeled a mosaic ommatidium in which the *N-cadherin* transgene is switched on in two neighboring P cells. Although this switching occurs at the same time in the two cells, the N-cadherin is unable to pair because the two P cells are not yet neighbors (cf. Fig. 8A). By the time they do become neighbors, the endogenous *N-cadherin* gene has been expressed in the cone cells for about 10 hours. Therefore, transgenic N-cadherin first pairs on the C1P and C2P edges (Fig. 8A), increasing adhesion and elongating these edges. The binding strength is *γ_N_^P^* = *βN_+_*/(2*L*_C2P_+*L*_C1P_), assuming that the P cells are limiting, which leaves *N_0_*−*N_+_ L*_C1P_/(2*L*_C2P_+*L*_C1P_) and *N_0_*−*N_+_ L*_C2P_/(2*L*_C2P_+*L*_C1P_) molecules in the C1 and C2 cells, respectively. The active interface lengths for the two different cone cell species are different (the C2 cells have to cover the center edge as well), but so are the numbers of cadherins to be distributed. The most important feature of the simulation is that, up to *N_+_*/*N_0_*≈1.2, the PP edges do not have paired N-cadherin at all, and therefore have a relatively high tension, leading to a characteristic acute P/C2/P angle seen in the simulation (Fig. 8B). Strikingly, an experimental mosaic ommatidium with misexpression in two P neighbors shows a similar morphology (Fig. 8D). A penalty function of the simulation compared to experimental showed a well-defined minimum at *N_+_*/*N_0_*≈1.2 (Fig. 9A–C). While the best-fit simulation yielded the cruciform shape with the acute P/C2/P angles seen in experiment, any significant deviation from this optimal *N_+_*/*N_0_* led to large shape changes. Note that this optimal ratio is nearly the same as that obtained from the simulation for the misexpression mutant in Fig. 7. The N-cadherin coverages of the various interfaces are plotted in Fig. 9D; note that any abrupt change in slope of the curves indicates a change in the sequence of limiting cells described above.

To determine if the simulation worked because of the timing of gene expression with respect to cell contact, we performed a simulation that disregarded the ordered cell contacts and assumed the P cells are in contact when *N-cadherin* expression begins. Since transgenic *N-cadherin* is expressed first, it would only be distributed along the PP edges, i.e., the binding strength there would be a large value *γ_N_^PP^* = *βN_+_*/(2*L*_PP_), and the effective tension on these edges quite small. The effect of this change produces a simulation that poorly fits the experiment (Fig. 8C). This failure was seen if N-cadherin was expressed either simultaneously or sequentially from the transgene and endogenous gene (Fig. 9A).

## Discussion

We have applied and extended the mechanical energy functional modeling on the morphology of *Drosophila* eye tissue in several crucial ways. This model relies entirely on passive global minimization of a simple interfacial energy functional. However, we have shown how to incorporate new features that, implicitly, describe observed biological features of the tissue. These features include: recycling and redistribution of unpaired N-cadherin molecules from the surface of cells, the temporal dynamics of N-cadherin gene expression, and the temporal dynamics of cell-cell contacts. Note that we established these model features without any additional free parameters. Because the dynamics of morphogenesis and gene expression are experimentally described phenomena, and because the Surface Evolver simulations are capable of varying these features, we incorporated these phenomena into a model that describes the experimental data with a high degree of accuracy. When we incorporate features into the model that are not observed in the tissue, the model does not simulate the experiments to the same degree. Two points can be made. First, it is essential for any morphogenesis model to be flexible enough that critical biological features can be modified. Second, our morphogenesis model not only simulates normal morphology but also morphologies observed in complex genetic mutants. In fact, it is the asymmetries in ommatidial shape and the cadherin expression dynamics introduced through mutants that allowed us to successfully test these features within the framework of the model.

The success of these simulations suggests that models employing energy minimization can go beyond the quantitative computation of local equilibrium morphologies, and become useful tools for simulating morphogenetic changes that are quasistatic with respect to the time scale of mechanical equilibration. In the case of changes in gene expression or cell neighbor relations, this condition is fulfilled, as these changes can take hours [41], [42], [43]. In contrast, mechanical equilibria are established in minutes at most, depending on dissipative effects [44], [45]. In the experiments we have described, there is also sufficient separation between the onset of cadherin expression and the changes in cell-cell contacts.

We simulated misexpression of a *N-cadherin* transgene to test many of the biological features of the model. In all of these simulations, the best-fit solutions predicted a level of transgenic *N-cadherin* expression level to be 1.2-fold greater than that of the endogenous *N-cadherin* gene. One would expect that independent simulations predict the same level of transgene expression because its expression is independent of which cell or combination of cells express it. Also encouraging is that the level of transgene expression is predicted by simulations to be comparable to the level of endogenous *N-cadherin* gene expression. The transgene uses the transcriptional promoter from the *actin5c* gene to indirectly drive transcription of the *N-cadherin* coding region [5]. Actin5c is one of six actin isoforms and represents only 5–10% of the actin in pupal head cells [46]. Thus, we estimate that 50,000 to 100,000 actin5c protein molecules would be present in these cells, and presumably a similar number of heterologous proteins produced from an *actin5c* transgene. About 50,000 endogenous cadherin protein molecules are present in a typical animal cell [47]. Therefore, it is reasonable to find that transgenic and endogenous production of N-cadherin are not different by orders of magnitude.

Most computer models of tissue morphogenesis simulate visual changes in cell shape and neighbor relations that occur over time [48]. However, use of qualitative visual information to compare normal and perturbed morphogenesis limits their abilities to find optimum mechanical parameters. Our model introduces a novel method for exploring morphogenesis: the effect of perturbations during morphogenesis can be simulated by the final cell shapes and neighbor relations that are achieved at the end of morphogenesis. The simplicity of the final cell geometry allows for unambiguous quantitative comparisons between different morphogenetic conditions, particularly for highly ordered tissues like the *Drosophila* retinal epithelium.

## Model

In previous work, we introduced a quantitative simulation method for normal cell geometry in the two-dimensional plane of the AJ based on two free parameters encoding for the binding strengths of E- and N-cadherin. Quantitatively, we minimized the mechanical energy functional (see equation (1))
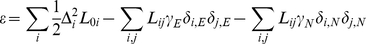
using the Surface Evolver software [49]. In (1), dimensional lengths *L^dim^* are normalized by a scale *L_S_* = *D*/9 (making *L_S_*≈1 µm), so that *L = L^dim^*/*L_S_*. Likewise, energies *E^dim^* are non-dimensionalized as *ε* = *E^dim^*/*E_S_*, where *E_S_* = *K_A_ b L_S_*. Here, *K_A_* is a uniform membrane stretch modulus (an energy per area), assumed uniform for all cell membranes [13], [50]. Note that all results we report are independent of the numerical values of *b*, *L_S_* and *E_S_*, as we will describe shapes through relative errors of the energy functional only.

Further, Δ*_i_* = *(L_i_−L_0 i_)/L_0 I_* is the strain [51] on the membrane of cell *i*, whose total circumference in the two-dimensional projection of the AJ is *L_i_*. At unstressed equilibrium (measured experimentally for cells detached from their neighbors), the circumference is *L_0 i_*. We scale the specific cadherin binding energies Γ*_E_* and Γ*_N_* by *K_A_* to obtain the dimensionless quantities *γ_E_* = Γ*_E_/K_A_* and *γ_N_* = Γ*_N_/K_A_*, which are the only parameters in the model. If the same cadherin is active in two neighboring cells *i* and *j* (note the Kronecker delta functions), the resulting binding energy is proportional to *L_ij_*, the length of the membrane between the cells. The minimization of (1) is performed under constraints of constant cross-sectional areas of the central cells in the ommatidium, formalized through Lagrange multipliers in Surface Evolver [6]. Tests incorporating either bending energy terms or a finite energy penalty for changes in cross sectional area did not yield significantly different results (see [6] for more details).

We arrived at unique values for *γ_E_* and *γ_N_* by quantitatively comparing experimental geometry data of normal ommatidia with those determined from our computational simulations minimizing (1), cf. [6]. All lengths and angles characterizing the observed ommatidium structure were reproduced within experimental error. In order to quantify the deviations between experimental and simulated structures, we summed up relative errors of both the lengths of edges and the angles between edges in the structures. Minimization of the total error function then yielded the best-fit wild-type values *γ_E_* = 0.025·0.005 and *γ_N_* = 0.032·0.005, respectively, where the errors reflect uncertainties in the geometrical data [6].

### Quantifying modeling errors

In order to have a more general measure for shape differences, we generalized the penalty function of [6], taking into account errors in (i) the interface lengths in the 2D structure, (ii) the angles between interfaces, and (iii) in cases where the entire unit becomes asymmetric, a measure of that global asymmetry. The latter quantity, *D*_x_, is the dimensionless distance of the PP edges to the nearest vertex of the hexagonal ommatidial frame (see Fig. 2; note that in a symmetric ommatidium with centered PP edges *D*_x_ = 1/4). The angles between edges (e.g. *θ_j_*, *θ_k_*, *θ_l_* in Fig. 2) are a measure of the differences in the mechanical tensions *τ_j_*, *τ_k_*, *τ_l_* along these edges (if all tensions are equal, all angles are 120·). In fact, the ratio of any two tensions is given by
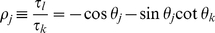
(3)and corresponding cyclic permutation of the indices (*jkl*). Even subtle differences in angles can translate into strong differences in tension ratios. Therefore, we adopt the quantities *ρ_l_* instead of the angles to obtain a more sensitive error measure.

Any quantity *X* contributes to the total error via a squared relative error,
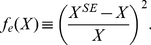
(4)


Here, the superscript SE denotes the quantity obtained from Surface Evolver simulations, while *X* without superscript is the experimental value. Our total error function is the sum of all *f_e_*. Explicitly,
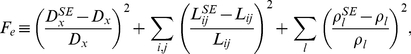
(5)where the indices *i,j* denote cells and the indices *l* denote angles. The smaller *F_e_*, the better is the agreement between the measured quantities and those obtained by simulations. In many cases, shape differences are dominated by only one or a few *f_e_*, while in others the complete set has to be included in *F_e_*.
