# Correction: Cadherin-Dependent Cell Morphology in an Epithelium: Constructing a Quantitative Dynamical Model

**DOI:** 10.1371/annotation/7bdb973d-e73b-42dc-878f-4681dea876f1

**Published:** 2011-08-15

**Authors:** Ian M. Gemp, Richard W. Carthew, Sascha Hilgenfeldt

There were errors in the Author Contributions. The correct contributions are: Conceived and designed the experiments: RWC. Analyzed the data: IMG SH. Contributed reagents/materials/analysis tools: SH. Wrote the paper: IMG RWC SH. Performed Numerical Simulations: IMG. Developed theory: IMG RWC SH.

